# Recent Advances in the Fabrication and Environmental Science Applications of Cellulose Nanofibril-Based Functional Materials

**DOI:** 10.3390/ma14185390

**Published:** 2021-09-17

**Authors:** Lianming Zhang, Lei Guo, Gang Wei

**Affiliations:** 1 School of Resources and Environmental engineering, Shandong Agriculture and Engineering University, Jinan 250100, China; lianmingzhang@163.com; 2 Institute of Biomedical Engineering, College of Life Science, Qingdao University, Qingdao 266071, China; 3 College of Chemistry and Chemical Engineering, Qingdao University, Qingdao 266071, China

**Keywords:** cellulose nanofibrils, functionalization, hybrid nanomaterials, environmental science, water purification

## Abstract

Cellulose is one of the important biomass materials in nature and has shown wide applications in various fields from materials science, biomedicine, tissue engineering, wearable devices, energy, and environmental science, as well as many others. Due to their one-dimensional nanostructure, high specific surface area, excellent biodegradability, low cost, and high sustainability, cellulose nanofibrils/nanofibers (CNFs) have been widely used for environmental science applications in the last years. In this review, we summarize the advance in the design, synthesis, and water purification applications of CNF-based functional nanomaterials. To achieve this aim, we firstly introduce the synthesis and functionalization of CNFs, which are further extended for the formation of CNF hybrid materials by combining with other functional nanoscale building blocks, such as polymers, biomolecules, nanoparticles, carbon nanotubes, and two-dimensional (2D) materials. Then, the fabrication methods of CNF-based 2D membranes/films, three-dimensional (3D) hydrogels, and 3D aerogels are presented. Regarding the environmental science applications, CNF-based nanomaterials for the removal of metal ions, anions, organic dyes, oils, and bio-contents are demonstrated and discussed in detail. Finally, the challenges and outlooks in this promising research field are discussed. It is expected that this topical review will guide and inspire the design and fabrication of CNF-based novel nanomaterials with high sustainability for practical applications.

## 1. Introduction

Rapid population growth, modern industrial development, and increased agricultural production have led to the pollution of natural and drinking water systems, in which a lot of toxic and harmful pollutants, such as metal cations, anions, oils, organic dyes, drug molecules, bacteria, and viruses, exist. Therefore, the development of novel techniques and materials for water purification has attracted great attention in the last years [1,2].

Other than the traditional water purification techniques of flocculation, polymer membrane filtration, reverse osmosis, and electrodialysis, the design and synthesis of high-performance nanomaterials with high adsorption capacity have great importance in water purification [3,4,5,6]. Cellulose is one of the most important and abundant biomass materials in nature, which can be extracted from natural biomass, including wood, leaves, bamboo, and others. Due to its large amount, low cost, high biocompatibility, good biodegradability, and potential sustainability, cellulose materials have been widely used in materials science, biomedicine, tissue engineering, wearable devices, energy and environmental science, as well as many other fields [7,8].

Compared to cellulose, cellulose nanofibrils/nanofibers (CNFs) have many advantages, such as a higher ratio of length to diameter, higher ability for hybridizing with other materials, larger specific surface area, easier operation, and feasibility to form fiber network for hydrogels/aerogels. For instance, CNFs have a one-dimensional (1D) nanostructure, which shows a larger specific surface area (it is much easier to apply for the fabrication of hybrid nanomaterials for nanotechnology applications [9,10]). In addition, CNFs have the characteristic to crosslink via physical and chemical interactions to form two-dimensional (2D) films/membranes and three-dimensional (3D) hydrogels/aerogels [11,12,13]. The fabricated CNF-based 1D, 2D, and 3D materials have shown promising applications for the adsorption and removal of various pollutants from water [14,15]. Previously, a lot of reviews on the synthesis, functionalization, and applications of cellulose and CNFs have been released [16,17,18,19,20]. For example, Jonoobi et al. summarized the preparation methods and properties of cellulose nanocrystals and CNFs, which were focused on the methodologies on the synthesis of nanostructured cellulose from natural resources [16]. Tapia-Orozco et al. summarized the strategies for the removal of endocrine-disrupting chemicals by cellulose-based adsorbents [17]. Du and co-workers presented a review on the fabrication of cellulose nanocrystal- and CNF-based hydrogels for biomedical applications [18]. Very recently, the preparation, modification, fabrication, and applications of cellulose-based aerogels have been presented comprehensively [21]. In addition, recently, the fabrication of nanocellulose-based bioadsorbents for chemical contaminant remediation [22] and water purification [23] and environmental remediation [24] has been presented. Although great achievements have been obtained, it is necessary to present a summary of the methodologies of CNF-based 1D, 2D, and 3D materials, and to demonstrate their applications in environmental science based on their tailored structure and function.

In this review, we focus on the synthesis and functionalization of CNFs for the fabrication of functional nanomaterials for environmental science applications. In Part 2, the strategies on the hybridization of CNFs with other nanocomponents, such as nanoparticles (NPs), quantum dots, metal-organic frameworks (MOFs), and carbon nanotubes (CNTs), are introduced. In addition, the fabrication of 2D CNF films and membranes via vacuum filtration and electrospinning, as well as the construction of 3D CNF hydrogels and aerogels, are summarized and discussed. In Part 3, we present recent progress on the synthesized CNF-based adsorbents for removing cations, anions, oils, dyes, and bio-contents. Compared to previous reviews, this work reveals the novelty and significance of both structural adjusting and functional tailoring of CNF-based functional materials and their emerging environmental science applications for removing various contaminants from water. This review will be helpful for readers to understand the synthesis, functionalization, hybridization, and 2D/3D construction of CNFs, and extend the applications of CNF materials in materials science, energy storage, biomedicine, tissue engineering, analytical science, and others.

## 2. Fabrication of CNF-Based 1D, 2D, and 3D Functional Materials

In order to promote the easy operation and application of CNFs, it is necessary to carry out suitable treatments on CNFs to form 2D and 3D nano/micro-structures. In this part, we present various strategies for the fabrication of CNF-based 1D CNF hybrids, 2D membranes/films, as well as 3D hydrogels and aerogels.

### 2.1. CNF-Based 1D Nanomaterials

Electrospinning and chemically synthesized CNFs can serve as nanoscale templates for the preparation of various CNF-based 1D nanomaterials. The surface of CNFs could be functionalized first with suitable chemicals and then achieved in combination with nanoparticles [25,26,27,28,29,30], quantum dots [31,32], metal-organic frameworks (MOF) [32,33], carbon nanotubes (CNTs) [34], and others.

**Figure 1:**
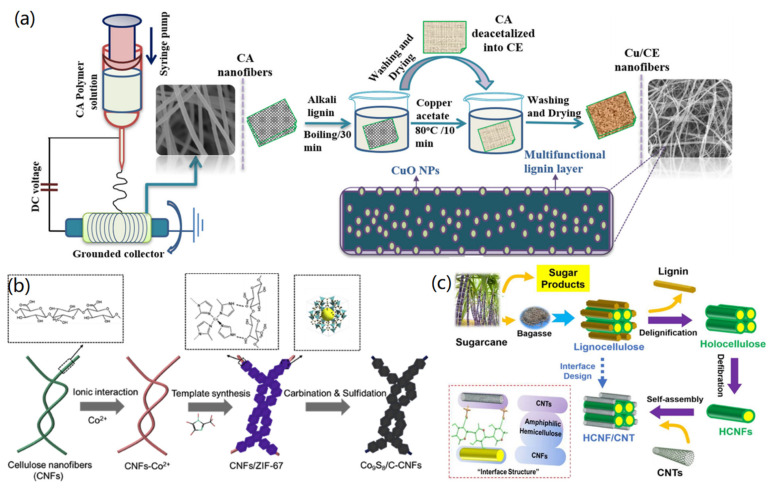
Synthesis of CNF-based 1D hybrid materials: (**a**) CNF/CuO hybrids. Reprinted with permission from Ref. [25], Copyright 2021, Elsevier Ltd. (**b**) CNF/MOF hybrids. Reprinted with permission from Ref. [33], Copyright 2020, Elsevier Ltd. (**c**) HCNF/CNT hybrids. Reproduced with permission from Ref. [34], Copyright 2020, American Chemical Society.

For instance, very recently, Haider et al. reported the in situ synthesis of CuO nanoparticles on CNFs [25], as presented in Figure 1a. Firstly, cellulose acetate (CA) was electrospun to form CA nanofibers, which were then reacted with alkali lignin and copper acetate to form CuO/CNF hybrids after washing and drying. The obtained results indicated that the bound alkali lignin improved the antioxidant activity of CNFs, and the in situ synthesized CuO nanoparticles improved the performance against both E. coli and S. aureus bacteria, showing the potential application of the designed CuO/CNF materials for wound dressing. With the similar methods, various nanoparticles, such as Ag [26,29,30], TiO_2_ [27], Ag@Au [28], and Cu [29], have been synthesized on CNFs for different applications. In addition to NPs, the quantum dots, such as carbon dots [31] and black phosphorus quantum dots [32], have been anchored onto the surface of CNFs to form 1D CNF fluorescent nanomaterials.

Alongside NPs and quantum dots, some other functional nanomaterials, such as MOFs and CNTs, can also be conjugated onto CNFs in order to enhance their functions and extend their applications. In a typical study, Guo and co-workers reported CNF-templated synthesis of ZIF-67/CNF composites for lithium storage application [33]. As shown in Figure 1b, Co^2+^ was added into a CNF solution to form CNFs-Co^2+^ composites via the ionic interaction, which then served as templates for the synthesis of CNFs/ZIF-67 under the addition of 2-methylimidazole to this reaction system. CNFs played crucial roles in controlling the growth of MOF particles, avoiding the aggregation of materials, and serving as conductive linking between ZIF-67 particles after carbonization and sulphidation of CNFs/ZIF-67 composites. In another study, Zhang and co-workers synthesized high-performance CNF/CNT nanocomposites through the interface design for flexible liquid sensing, as shown in Figure 1c [34]. Via the self-assembly and design of interface interactions between pre-synthesized holocellulose nanofibrils (HCNFs) and CNTs, amphiphilic HCNF/CNT composites with high tensile strength (121 MPa), high relative humidity (83%), and good electrical conductivity (321 S/m) have been produced successfully.

### 2.2. CNF-Based 2D Films/Membranes

Two-dimensional film and membranes have large specific surface areas, porous network structures, and a large amount of active sites for the adsorption of various cations, anions, dyes, oils, and other biomolecules. Therefore, the fabrication of CNFs into 2D films/membranes can make the material easy to operate and applicable for many fields. In general, the fabrication strategies of CNFs to 2D films/membranes can be summarized as techniques such as vacuum filtration, electrospinning, freeze-casting, and room temperature casting.

**Figure 2:**
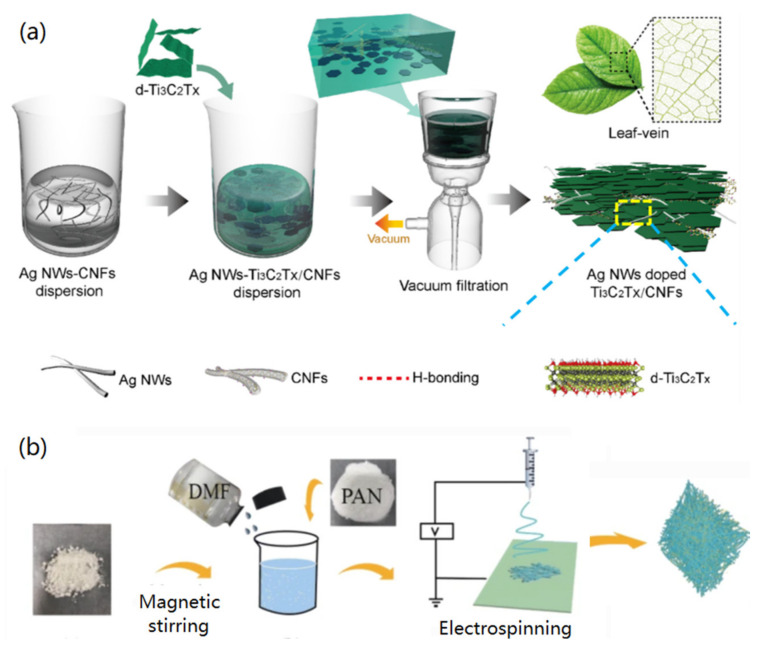
Fabrication of CNF-based 2D films/membranes via (**a**) vacuum filtration, reprinted with permission from Ref. [37], Copyright 2021, Elsevier Ltd., and (**b**) electrospinning, reproduced with permission from Ref. [38], Copyright 2020, Springer.

Vacuum filtration has been widely used for the fabrication of various membrane materials on a filter through physical deposition materials. Previously, porous thin film/membrane of 2,3-carboxylic acid-modified CNFs [35] and CNFs/metal-organic-framework hybrids [36] have been fabricated via the vacuum filtration technique successfully. In a typical study, Liu et al. demonstrated the fabrication of ultrathin, biomimetic CNF-based nanocomposite film by the vacuum filtration of the mixed silver nanowires (Ag NWs)-Ti_3_C_2_T_x_/CNFs dispersion (Figure 2a) [37]. The microscopy measurement indicated that the obtained hybrid film surface was formed by tightly stacked Ti_3_C_2_T_x_ nanosheets and exhibited a leaf-like morphology. By the hydrogen bonding and electrostatic interaction, the presence of CNFs promoted the linking between Ti_3_C_2_T_x_ and AgNWs to form multifunctional films.

Electrospinning is one of the facile and practical techniques in nanotechnology and materials science for the fabrication of nanofiber membrane materials, which has the advantages of simple preparation, low cost, controllable process, and flexibility for various precursor materials. For the preparation of 2D CNF-based films and membranes, CNFs are firstly mixed with a polymer solution to form polymer/CNF hybrid membranes via electrospinning. Xu and co-workers reported the fabrication of polyacrylonitrile (PAN)/CNF hybrid membranes using electrospinning [38]. As shown in Figure 2b, CNFs were dissolved in DMF solution, and PAN was added under magnetic stirring to get the electrospinning precursor solution, which was then electrospun to form PAN/CNF hybrid membranes on a solid collector. In other cases, poly(lactic acid)/CNF [39] and polymer/bacterial cellulose [40] hybrid membranes have been produced via a similar electrospinning method. This technique has been widely utilized for the fabrication of various biomass-based membrane materials in recent years. In addition, after the carbonization of the formed hybrid organic nanofibers, it is possible to get carbon nanofiber-based materials with enhanced applications [41,42].

In addition, the casting of the CNF solution could be another strategy for the fabrication of CNF-based films/membranes. For instance, Pottathara et al. reported the preparation of oxidized CNFs/GO composite films by solution casting [43]. The created films had open-pore capillary microstructure, one-direction arrangement, a large surface-to-volume ratio, and a high adsorption capacity. The control experiment indicated that the introduction of GO to the composite films enhanced the adsorption capacities by 100–200 times that of pure CNF films. In the other case, the freeze-casting method has been utilized for the fabrication of CNF films [44] and hybrid carboxymethyl cellulose (CMC)/CNF assembled membranes [45].

### 2.3. CNF Hydrogels

Previously, hydrogels have been widely used in biomedicine, tissue engineering, food science, and wearable devices [18,46]. In a hydrogel system, the dispersion phase is normally water, and the solid phase has a 3D network structure of crosslinked materials. It has been proven that hydrophilic CNFs with charged interfaces are beneficial to fabricate hydrogels with high stiffness, high toughness, and high tensile strength, in which CNFs could serve as nanoscale building blocks and reinforcing nano-agents to form functional hydrogels. In this section, we would like to introduce several typical methods for mediating the crosslinking and gelation of CNFs for the formation of hydrogels.

**Figure 3:**
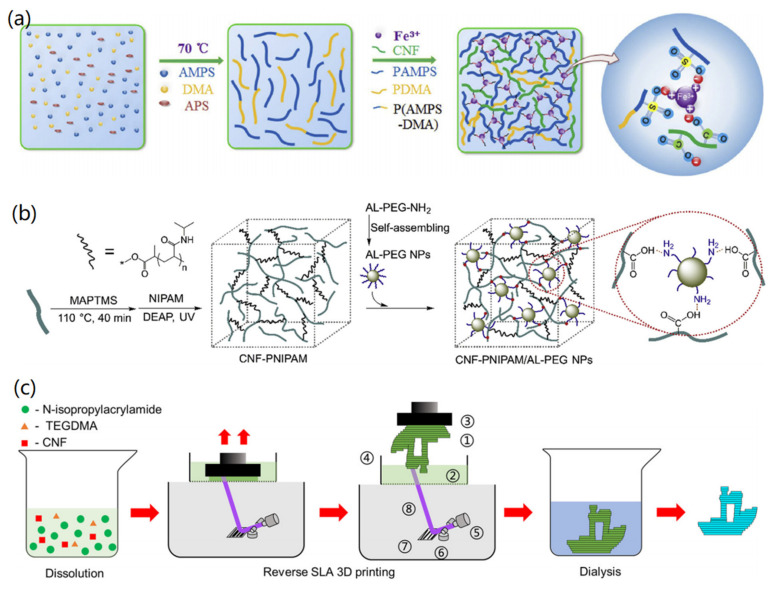
Fabrication of CNF-based hydrogels: (**a**) Fe^3+^-mediated crosslinking and CNF hydrogel formation. Reprinted with permission from Ref. [49], Copyright 2019, Elsevier Ltd. (**b**) Polymer-mediated self-assembling and CNF hydrogel formation. Reprinted with permission from Ref. [51], Copyright 2019, Elsevier Ltd. (**c**) 3D printed PNIPAm/CNF hydrogels. Reprinted with permission from Ref. [52], Copyright 2020, Elsevier Ltd.

Firstly, metal cations, such as Fe^3+^ and Zn^2+^, can mediate the crosslinking of CNFs and the formation of CNF hydrogels [47]. For instance, Lu et al. reported the preparation of CNF hydrogels induced by Zn^2+^ ions [48]. The gelation was based on the strong crosslinking between negatively charged carboxylate groups of CNFs and Zn^2+^ ions. The formed CNF hydrogels had the potential application for colorimetric monitoring of food spoilage in food packaging. In another study, Liu et al. presented that Fe^3+^ could mediate the crosslinking reaction between CNFs and two polymers, 2-acrylamido-2-methylpropane sulfonic acid (AMPS) and N,N-Dimethylacrylamide (DMA), as shown in Figure 3a [49]. At 70 °C, the addition of APS induced the polymerization of AMPS and DMA to both PAMPS and P(AMPS-DMA). After the introduction of CNFs and Fe^3+^, the electrostatic interaction between Fe^3+^ and negatively charged carboxylate and sulfonic acid groups promoted the crosslinking of CNFs and polymers, resulting in the gelation of CNFs.

CNFs can be used as reinforced nano-agents for the fabrication of hybrid hydrogels [50,51]. Aarstad et al. reported that CNFs could reinforce the alginate content to form composite hydrogels with high strength, high compressibility, and small deformation [50], and therefore, could be useful for tissue engineering and regenerative medicine applications. Lu and co-workers demonstrated the synthesis of CNF-reinforced polymer hydrogels through self-assembling and -COOH/NH_2_ interaction [51]. The polymerized AL-PEG NPs have rich -NH_2_ groups, which could create ionic bonds with -COOH groups of CNFs, resulting in a porous, interconnected, network structure of hydrogels with thermo- and pH-responsive properties.

Alongside the chemical gelation of CNFs into hydrogels, physical methods, such as 3D printing [52] and ink writing [53], can also be used for the fabrication of CNF hydrogels. These methods are based on the characteristics of both solution and instrument, which are able to create CNF hydrogels with various shapes. For instance, Sun and co-workers demonstrated the synthesis of PNIPAm/CNF hybrid hydrogels via 3D printing (Figure 3c). The formed 3D printable hydrogels had thermo-responsive properties and tunable bioadhesive behaviors, showing temperature-sensitive antibacterial properties [52]. In addition, mechanical methods, such as high-pressure homogenization and refining, were typical techniques for creating nanoscale and microscale CNFs, by which CNFs with adjusting size and morphology, as well as enhanced bonding potential, could be produced [54,55]. Through the homogenization and refining of cellulose, it is also possible to promote the crosslinking and binding of created CNFs for the formation of hydrogels with highly tangled fibril networks [56,57].

Many cases on the fabrication of cellulose- and CNF-based hydrogels have been reported previously. To get more understanding on synthesis, structure, properties, and applications of CNF-based hydrogels, the readers are suggested to study one recent review by Du and co-workers [18].

### 2.4. CNF Aerogels

After the formation of hydrogels, further treatment methods, such as freeze-thawing/drying and freeze-drying, can be applied to obtain aerogels. Freeze-casted CNF aerogels had lamellar pores, highly oriented structure, ultralightweight, high water adsorption capacity, and good shape-memory properties, showing wide applications in energy storage, pollutant adsorption, drug delivery, tissue engineering, wearable devices, and space materials [58,59,60].

**Figure 4:**
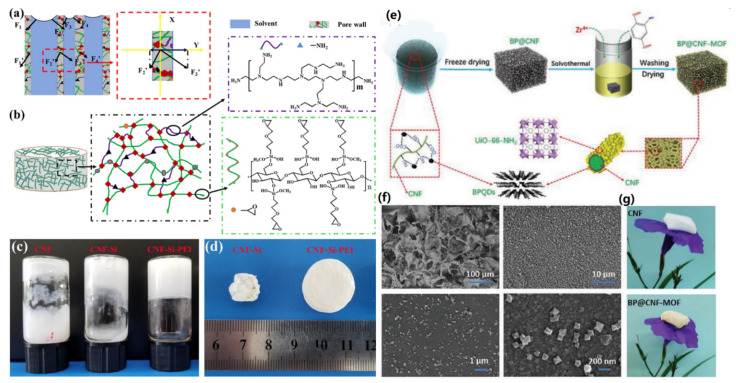
Fabrication of CNF-based aerogels. (**a**–**d**) CNF and CNF/PEI aerogels: (**a**) stress analysis, (**b**) cross-inking for the formation of aerogels, (**c**) gels after freeze-thawing, and (**d**) aerogels after drying. Reproduced with permission from Ref. [61], Copyright 2019, Elsevier Ltd. (**e**–**g**) BP@CNF-MOF aerogels: (**e**) synthesis strategy, (**f**) SEM characterizations, and (**g**) photographs of CNF and BP@CNF-MOF aerogels. Reproduced with permission from Ref. [32], Copyright 2021, Wiley VCH.

Li and co-workers presented the construction of crosslinked CNF aerogels via the freezing and atmospheric drying processes [61]. Figure 4a shows the stress analysis on the pore walls of the created porous hydrogel materials under the drying process. It was concluded that strengthening the pore network structure and decreasing the surface tension of the solvent were two simple ways to fabricate CNF aerogels via atmospheric drying. In their work, glycidoxypropyltrimethoxysilane (GPTMS) and polyethyleneimine (b-PEI) were used to crosslink CNFs (Figure 4b) to form hydrogels, in which the silicon hydroxyl groups from GPTMS could bind covalently with the hydroxyl groups of CNFs, and the epoxide groups of GPTMS could react with the amine groups of b-PEI to form higher density network structure. After freeze-thawing, the CNF-Si-PEI suspension revealed gel-like properties, but the CNF suspension was still in a liquid state (Figure 4c). Due to the strong crosslinking between CNF and GPTMS, the CNF-Si-PEI aerogels did not show structure collapse after atmospheric drying (Figure 4d). In another case, Toivosen et al. also fabricated CNF aerogels via freezing and ambient drying [62], which were highly tensile, transparent, and flexible for wearable device applications.

Direct freeze-drying can also be applied for the fabrication of CNF-based aerogels [63]. As shown in Figure 4e, Chen and co-workers reported the fabrication of CNF aerogels via the freeze-drying technique [32]. The formed CNF hydrogels served as templates for the anchoring of black phosphorus quantum dots (BPQDs), and BP@CNF aerogels were formed after freeze-drying treatment. After solvothermal synthesis, UiO-66-NH_2_ MOF was created on the CNF surface to form BP@CNF-MOF composites (Figure 4f). Finally, the BP@CNF-MOF aerogel was prepared via a washing and drying process. The formed aerogel exhibited porous structure and ultralightweight (Figure 4g), and can be used to adsorb and photocatalytically reduce U(IV) in water.

In order to make it more clear for the design and synthesis of CNF-based 1D, 2D, and 3D materials, here we provide a table (Table 1) to summarize the above-presented studies.

## 3. Environmental Science Applications

CNF-based functional materials have been widely used in various fields. In this section, we focused on the presentation and discussions of CNF-based materials in environmental science, including the highly effective removal of various heavy metal ions, anions, organic dyes, oils, and bio-contents.

### 3.1. Removal of Heavy Metal Ions

With the continuous development of current society, the requirements of the environment are increasing, in which the water pollution is a more serious problem in environmental protection, and how to effectively remove toxic heavy metal ions in water is an important scientific research direction and a problem that many scientists continue to study. In addition to having more surface activity sites, large surface area, and very high renewable capacity, CNFs have a higher strength after functionalization; these advantages make CNF-based nanomaterials become the best choice for the adsorption of heavy metal ions.

**Figure 5:**
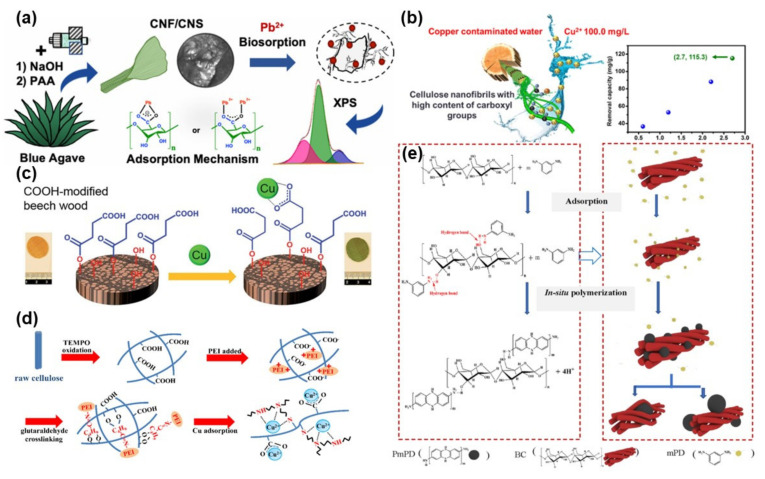
CNF-based nanomaterials for the removal of metal ions: (**a**) Blue agave-derived CNFs/nanosheets for removing Pb^2+^ ions. Reprinted with permission from Ref. [64], Copyright 2020, Springer. (**b**) Carboxymethylated CNFs for the removal of Cu^2+^. Reprinted with permission from Ref. [15], Copyright 2019, American Chemical Society. (**c**) Wood-derived CNFs for the removal of Cu^2+^. Reprinted with permission from Ref. [67], Copyright 2019, Royal Society of Chemistry. (**d**) TEMPO oxidization and PEI grafted CNFs for the removal of Cu^2+^. Reprinted with permission from Ref. [69], Copyright 2016, Elsevier Ltd. (**e**) PmPD/CNF materials for the removal of Cd(VI). Reprinted with permission from Ref. [71], Copyright 2018, Elsevier Ltd.

Hernández-Francisco et al. reported the preparation of native Mexican agave-derived CNFs/cellulose nanosheets to achieve the adsorption of divalent Pb^2+^ ions [64]. As shown in Figure 5a, the clean and dry blue agave was first alkali-treated, followed by microcurrent treatment to obtain cellulose nanofibers/nanosheets. The resulting cellulose nanofibers/nanosheets for adsorption testing indicated that the obtained cellulose nanofibers/nanosheets can be combined with Pb^2+^ ions via electrostatic interaction, thus achieving a good adsorption effect. Vadakkekara and colleagues used cellulose to react with maleic acid and then exchanged sodium ions to synthesize CNFs with carboxylic groups [65]. The functionalized CNFs had good adsorption capacity toward Pb^2+^ in water. In addition, the formed adsorbent could be regenerated by using NaCl solution, which was highly useful for the heavy metal ions adsorption system. In another case, the poly(methacylic) acid-*co*-maleic acid copolymer has been utilized to graft CNFs to form a reusable adsorbent for the removal of various metal ions, such as Pb^2+^, Cd^2+^, Zn^2+^, and Ni^2+^ ions [66].

The functionalization of CNFs can promote the applications of CNF-based nanomaterials in water purification, which attracted increasing attention recently. Qin and co-workers produced carboxymethylated CNFs, which could be applied to transfer the Cu^2+^-polluted water into drinking water directly [15], indicating their high adsorption ability to Cu^2+^ (Figure 5b). In a similar study, Hong et al. proposed the design of a modular adsorbent by embedding carboxymethylated CNFs in polyurethane foam, which combined the unique adsorption properties of both CNFs and polymer foam to achieve a superior high adsorption capacity and excellent recyclability [14]. In addition, Keplinger et al. have found that wood and wood-derived CNFs can be a very promising heavy metal adsorbent [67]. As indicated in Figure 5c, a highly porous layered wood CNF scaffold had a lot of evenly distributed carboxy groups, which exhibited high efficiency for the removal of Cu^2+^ ions from an aqueous solution. Although this research was still in the development stage, it guides and inspires the design and synthesis of green, high-performance, and expandable adsorbents for water purification applications.

TEMPO-mediated oxidation of CNFs can also improve the adsorption performance of CNF-based materials for the removal of metal ions. For instance, Lin demonstrated the formation of porous CNF beads using microwave-assisted TEMPO oxidation, which exhibited excellent adsorption ability for both metal ions and organic dyes [68]. Through microwave treating and TEMPO oxidation, CNFs maintained their original structure with a greater number of carboxyl groups; therefore, improving the adsorption ability of pollutants and extending the applications in industries. Zhang and co-workers found that the TEMPO oxidation and polyethyleneimine (PEI) grafting of CNFs could also improve the adsorption capacity toward Cu^2+^ [69]. As presented in Figure 5d, after TEMPO oxidation and PEI grafting, the number of carboxyls has increased significantly. Therefore, a lot of active sites for the adsorption of metal ions are created, resulting in a stronger ability to adsorb Cu^2+^ than pure CNFs. In another case, Kazim et al. demonstrated the synthesis of CNF-based poly(2-hydroxy ethyl methacrylate-glycidyl methacrylate) cryogels for the adsorption of Fe^2+^ ions [70]. With the addition of TEMPO-oxidized CNFs into the polymer, the adsorption amount of Fe^2+^ was increased from 23.07 (initial polymer adsorbent) to 400 mg/g (CNF-polymer cryogels).

Yang et al. combined the easy recovery characteristic of bacterial CNFs with the large adsorption capacity of poly(m-phenylenediamine) to create a functional CNF/PmPD hybrid material [71]. The interstitial phenylamine monomer, mPD, was in situ oxidized and polymerized on CNFs to form the stable hybrid CNF/PmPD nanoparticle materials, which exhibited strong adsorption capacity for Cd(VI) ions (Figure 5e). In another study, Wang and co-workers reported the preparation of a novel CNF-FeNP hybrid hydrogel for the adsorption of Cr(VI) [72]. The adsorption mechanism is based on electrostatic adsorption, reduction, and co-precipitation.

Aside from the above materials for the removal of metal ions from water, it is possible to prepare anionic CNFs for the adsorption of metal ions via electrostatic interaction. It has been reported that anionic CNFs could effectively absorb metal ions and sulfates in order to reduce the concentration of metal ions and acid content in acidic mining wastewater [73].

### 3.2. Removal of Anions

Alongside the adsorption of metal cations, CNF-based functional adsorbents can be used for the removal of anion pollutants, such as PO_4_^3−^, SO_4_^2−^, NO_3_^−^, F^−^, and I^−^. In some cases, suitable modifications of CNFs are needed to create positively charged CNF materials, which can further adsorb negatively charged anions through electrostatic interaction, ion exchange, and complexation.

**Figure 6:**
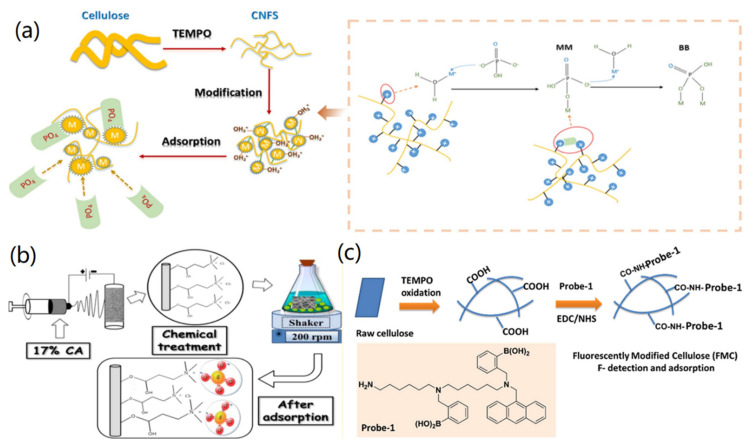
CNF-based adsorbents for the removal of anion ions: (**a**) Removal of PO_4_^3−^. Reprinted with permission from Ref. [76], Copyright 2016, Elsevier Ltd. (**b**) Removal of SO_4_^2−^. Reprinted with permission from Ref. [78], Copyright 2017, American Chemical Society. 2016. (**c**) Removal of F^−^. Reprinted with permission from Ref. [80], Copyright 2018, Elsevier Ltd.

Phosphate is an important natural resource, which has been exhausted rapidly. Therefore, the adsorption, removal and recovery of PO_4_^3−^ ions reveal high importance in many fields. To improve the electrostatic adsorption of cellulose-based materials on PO_4_^3−^ ions, previously the modification of cellulose with amino-rich polymer [74] and Fe(III)-coordinated amino-functionalized polymer [75] have been utilized. It has been proven that the introduction of amino groups and Fe(III) into cellulose materials enhanced the adsorption capacity of materials on PO_4_^3−^ ions. Based on the high binding affinity of ferric hydroxide (Fe(OH)_3_) towards PO_4_^3−^ ions, Cui and co-workers fabricated a novel hybrid adsorbent through the modification of CNFs with Fe(OH)_3_ for highly effective removal of PO_4_^3−^ ions from wastewater [76]. As shown in Figure 6a, cellulose was firstly oxidized with TEMPO into CNFs, which were then modified with Fe(OH)_3_ to form Fe(OH)_3_-coated CNFs (Fe(OH)_3_@CNFs) via the interaction between carboxylic acid groups and Fe^3+^ cations. In the adsorption process, PO_4_^3−^ ions were adsorbed onto Fe(OH)_3_@CNFs through electrostatic interaction and ligand exchange. Through Lewi acid-base interaction, the unprotonated O atoms of PO_4_^3−^ could form inner-sphere complexes by exchange with the -H_2_O and -OH groups of Fe(OH)_3_@CNFs, by which both monodentate mononuclear (MM) and bidentate binuclear (BB) complexes could be formed. The synthesized Fe(OH)_3_@CNFs exhibited a high adsorption capacity of 142.86 mg/g for PO_4_^3−^ ions. In addition, the removal operation can be carried out under neutral conditions, showing its potential application for treating phosphate-rich wastewaters with high efficiency, simple operation, and low cost.

Highly concentrated SO_4_^2−^ ions have strong negative effects on human health and the environment, including the corrosion of steel pipes, damage to mammals, inducing the mineralization of water, and formation of acidic H_2_S gas pollutants. To remove SO_4_^2−^ ions, Gao et al. fabricated cellulose derivative-based hybrid materials by combining carboxylate cellulose and ammonium-functionalized cellulose, which served as good biosorbents for the removal of Cu^2+^ and SO_4_^2−^ ions simultaneously [77]. In another case, Muqeet and co-workers reported the cationization of CNFs for the adsorption and removal of SO_4_^2−^ ions from water [78]. As shown in Figure 6b, CNF mats were prepared by electrospinning 17% cellulose acetate (CA), which were then cationized with 3-chloro-2-hydroxypropyl trimethylammonium chloride (CHTAC) to form cationized CNFs. It was found that the created cationized CNFs with 0.134 mmol/g ammonium content exhibited 24.5 mg/g adsorption capacity and a high adsorption rate of 0.0022 mg g^−1^ min^−1^ towards SO_4_^2−^ ions. The further adsorption mechanism study indicated that the high adsorption efficiency of CHTAC-grafted CNFs was based on the electrostatic interaction between positively charged ammonium groups of cationized CNFs and negatively charged SO_4_^2−^ ions.

The functionalization of CNFs can also improve the adsorption ability of CNF-based materials towards F^−^ ions. Previously, Yu et al. demonstrated that the in situ hybridized hydroxyapatite (HA) on cellulose fibers revealed a strong affinity towards F^−^ ions [79]. Due to the introduction of nanoscale HA into cellulose fibers, the designed hybrid material showed a high adsorption capacity toward F^−^ ions, which was not affected by the co-existing anions, such as the PO_4_^3−^, SO_4_^2−^, and NO_3_^−^ ions. In another case, the functionalization of CNFs with a fluorescent probe for the preparation of fluorescent modified cellulose (FMC) has been reported [80]. As shown in Figure 6c, raw cellulose was changed to CNFs via the TEMPO oxidization, which was then modified with EDC/NHS to covalently bind with fluorescent probe (Probe-1) to form FMC. It was found that the created FMC reveals dual functions for selective detecting and removing F^−^ ions. As different fluorophores could be utilized for the synthesis of functional FMC materials, this design strategy provides a facile way for the synthesis of CNF-based nanomaterials for anion sensing and removing applications.

In addition to the electrostatic adsorption and ion exchange with designed functional materials, the electrode-based adsorption has been utilized for the selective removal of anions from wastewater. In a typical study, Li and co-workers fabricated a NO_2_^−^ selective electrode by using cation-exchange polymers (glutaric acid and sulfosuccinic acid) to modify bacterial CNFs [81]. It was found that the formed electrode exhibited enhanced NO_2_^−^ selectivity and improved the adsorption capacity. In addition, with the increasing of applied voltage from 0.8 to 1.2 V, the total adsorbed anion amount was increased. It is interesting that this CNF-based electrode was useful for the removal of other anions, such as SO_4_^2−^, NO_3_^−^, F^−^, and Cl^−^ ions. However, it revealed high selectivity towards NO_2_^−^ > SO_4_^2−^ > NO_3_^−^ > F^−^ = Cl^−^. Further application of the electrode for real wastewater samples proved that it has excellent performance for treating dyeing wastewater.

### 3.3. Removal of Organic Dyes

Currently, the textile industries are the prime factor that causes water pollution due to a lot of unfixed dyes that are dispersed into the water resource during the dyeing process. Most of the organic dyes in water pollutants are highly toxic, non-degradable, and harmful to human beings and biological organisms. Therefore, the adsorption and removal of dyes from water have attracted more and more attention in the last years. CNF-based functional nanomaterials, such as pure CNFs [82], dried/pyrolyzed CNFs [83], positively/negatively modified CNFs [84,85,86], hybrid CNF/chitosan [87,88], and catalyst-modified CNFs [89,90,91], have also shown potential application for effective removal of organic dyes.

Previously, Purington et al. reported that CNFs could be utilized to adsorb fluorescent dye for the production of tagged CNFs [82]. Another study indicated that the drying and pyrolysis of CNFs that are produced from a few biomasses, such as wood, bacteria, and algae, could be used for oil absorption and dye adsorption [83]. For the adsorption of dyes, the created dried CNF chars exhibited large specific surface area, which was beneficial for the access of dye molecules into/onto the inner/outer parts of the materials. In addition, it was found that the drying of CNFs greatly enhanced their surface area and could remove 99.8% methylene blue solution with a concentration of 40 mg/L. The dried CNF materials had max adsorption capacities of 44–158 mg/g for the typical organic dyes, including methylene blue, crystal violet, and Congo red.

The modification of CNFs with positively and negatively charged chemicals can promote the adsorption of different dyes with high selectivity. For instance, Maatar et al. reported the modification of CNFS with cationic chemical glycidyltriethylammonium chloride to form microporous cationic CNF aerogels through freeze-drying technique [85]. The fabricated aerogel exhibited high adsorption ability for acidic and anionic dyes. In another study, Sehaqui and co-workers demonstrated the modification of cationic CNFs with humic acid, which could be applied for bioinspired removal of Cu^2+^ and positively charged dye, methylene blue hydrate [86].

**Figure 7:**
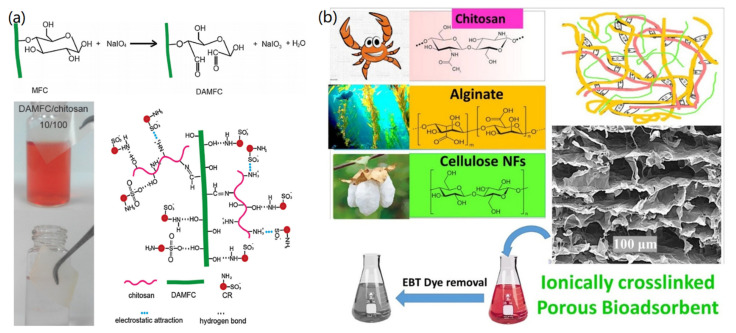
CNF-based functional nanomaterials for the removal of dyes: (**a**) Dialdehyde microfibrillated cellulose/chitosan film for removing Congo red. Reproduced from Ref. [87], Copyright 2018, Elsevier Ltd. (**b**) 3D porous chitosan/alginate/CNF bioadsorbents for removing Eriochrome black-T (EBT) anionic dye. Reprinted from Ref. [88], Copyright 2021, Elsevier Ltd.

In addition, CNFs can be combined with other biomass, such as chitosan and sodium alginate, to form hybrid 2D films and 3D aerogels for removing dyes from water. Previously, dialdehyde microfibrillated CNFs (DAMFC) were used to conjugate with chitosan to form DAMFC/chitosan composite film by the solvent-casting technique [87], as shown in Figure 7a. Due to the formation of network structure and the addition of DAMFC, the formed hybrid film exhibited improved adsorption performance toward the removal of Congo red, with a high removal efficiency of 99.95% and adsorption capacity of 152.5 mg/g. Based on the obtained results, the adsorption driving forces were proposed to be both electrostatic attractions between -NH_3_^+^ and Congo red as well as the hydrogen bond between -OH and Congo red (Figure 7a). In another case, bioadsorbents were fabricated by freeze-drying chitosan/alginate/CNFs together into a 3D porous, interconnected structure, which also revealed a high adsorption capacity of 2297 mg/g toward Eriochrome black-T (EBT), as shown in Figure 7b [88]. It was clear that the fabricated biomass-combined CNF film and 3D scaffold preserved high stability and adsorption capacity. In addition, the designed materials were economic, green, recyclable, and biodegradable, showing high sustainability in the purification of dye-polluted water.

Aside from the adsorption-based removal of dyes, it is possible to remove dyes by improving the catalytic oxidization activity of CNF-based materials. For instance, cobalt tetraaminophthalocyanine (CoPc) and MnO_2_ have been applied to modify CNFs to form CoPc@CNF [89], MWCNT/CoPc@CNF [90], and MnO_2_@CNF [91] nanocomposites with high catalytic activity, which showed potential decoloration capacity toward Rhodamine B, reactive red X-3B, and methylene blue, respectively.

In industrial production, typical dyes, such as methyl orange and phenol red, have been applied widely. Cellulose and CNF-based materials have been synthesized to adsorb and remove these dyes [92,93,94,95]. For instance, Hasanpour et al. reported the synthesis of cellulose/ZnO hybrid aerogels for the efficient removal of methyl orange, and the removal efficiency could reach 99.02% after 90 min of treatment [93]. Narwade and co-workers demonstrated the in situ synthesis of CNF/hydroxyapatite hybrid films for the removal of phenol from wastewater [95]. The removal amount of phenol from acidic and neutral solutions was about 64 and 30 mg/g, respectively.

### 3.4. Oil Removal

Spilled oil causes a great threat to the natural environment, and oil pollution in water and soil systems could be solved with polymer porous materials and 3D aerogels [96,97]. CNF-based nanocomposites, membranes/films, and aerogels have been utilized as environmentally friendly materials for the removal of oils.

In most cases, CNF-based materials are used as highly effective adsorbents for the removal of oils. Fu et al. demonstrated the preparation of strong, mesoporous, and hydrophobic cellulose/epoxy biocomposite using wood nanotechnology [98]. The formed biocomposites showed oil absorption as high as 15 g/g. It is interesting that the biocomposites could absorb oil from below and above the oil/water surface due to the hydrophobic and mesoporous structure of the cellulose-based composites.

In addition to CNF-based nanocomposites, CNF-based 2D membranes and films can be utilized for the removal of oils. For instance, Kollarigowda et al. reported the fabrication of CNF-based superhydrophobic hybrid biomembranes for effective oil/water separation [99]. For this aim, the hydrophilic CNF membranes were modified with a hydrophobic block copolymer, poly(3-(trimethoxysilyl)propyl acrylate)-block-myrcene, to form a superhydrophobic surface via a polymerization reaction. The adsorption test indicated that the fabricated superhydrophobic membranes exhibited superior extractive activity and high reusability for oil/water separation. In addition, the CNF-based biomembranes revealed a high ability to inhibit the polluted water by serving as an excellent antifouling material. Recently, Zhu and co-workers reported the fabrication of sandwich-like Ag-Pulp/CNF composite paper via an alternating filtration with Ag NP-coated pulp fibers and CNFs [100]. The obtained composite paper exhibited 96% separation efficiency for the oil/water separation, and the paper could be re-used for over 20 separation cycles. Ascribing to the action of Ag NPs, the fabricated composite paper revealed high antibacterial performance.

3D CNF-based aerogels and sponges have also been utilized for the separation of oils from the oil/water systems due to their large specific surface area, low density, porous structure, good biodegradability, and high adsorption ability. CNFs have been surface treated with hydrophobic styrene-acrylic monomer [101], silane solutions (20 wt%) [102], polydopamine/octadecylamine (PDA/ODA) [103], and oxidation-sulfonation [104] to form superhydrophobic CNFs, which were then freeze-dried to form functional CNF aerogels for oil/water separation applications.

**Figure 8:**
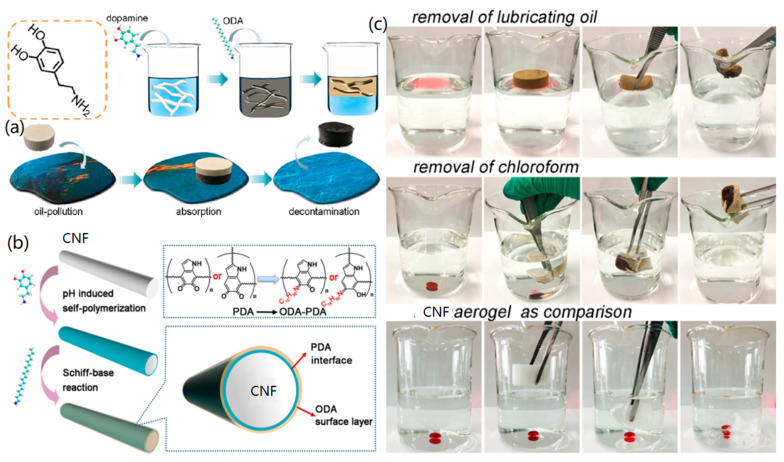
PDA/ODA-modified CNFs for the synthesis of aerogels for oil/water separation: (**a**) Fabrication of superhydrophobic CNF-based aerogels for the removal of oil, (**b**) surface modification of CNFs with PDA and ODA, and (**c**) separation the oil and chloroform from water. Reproduced with permission from Ref. [103], Copyright 2018, American Chemical Society.

For instance, Gao et al. demonstrated the mussel adhesive-inspired synthesis of superhydrophobic CNF aerogels for effective oil/water separation [103], as shown in Figure 8a. For this aim, CNFs were modified with PDA and ODA subsequently to form hydrophobic PDA-ODA@CNFs, which were then freeze-dried to form PDA-ODA@CNF aerogels. For the modification of CNFs, the binding of PDA was achieved through pH-induced self-polymerization, and the binding of ODA was carried out via the Schiff-base reaction, as indicated in Figure 8b. The fabricated CNF-based composite aerogel was ultralight (6.04 mg/cm^3^) and had a high contact angle (152.5°), endowing the formed aerogels with high oil/water separation selectivity. Due to the nanoporous structure of aerogels, oil could be absorbed into the aerogels from the oil/water mixture system. As presented in Figure 8c, the fabricated aerogels exhibited high performance for removing oil and organic solvents (such as chloroform). In addition, the comparison experiment indicated that the PDA-ODA@CNF aerogels had a higher adsorption ability than neat CNF aerogels. Sun and co-workers reported the modification of CNFs via the oxidation-sulfonation for the fabrication of superoleophobic CNF aerogel for oil/water separation [104]. After the treating, the formed CNF aerogel revealed enhanced hierarchical structure due to the effect of –SO_3_^−^ groups and resulted in superoleophobicity underwater. Therefore, in the oil/water mixture, the water phase could penetrate into the aerogel, but nonpolar oil was inhibited outside the aerogels for achieving the separation. In addition, it was found that the oxidation-sulfonation process greatly enhanced the separation efficiency and improved the stability for separation recycling.

To improve the mechanical properties of biomass aerogels, Yang and co-workers fabricated superelastic and highly hydrophobic/superoleophilic sodium alginate (SA)/CNF aerogels via different freeze-casting methods [105]. The bidirectional freezing on the fabrication of SA/CNF aerogels made the materials have parallel lamellar microstructure and superelasticity. In addition, the contact angles of water and oil on the surface of SA/CNF aerogels were 148.7° and O°, respectively, indicating their multifunctions. Therefore, the created SA/CNF aerogels could adsorb oil with a capacity up to 34 times the weight of aerogels.

### 3.5. Removal of Bio-Contents

Aside from the above-mentioned cations, anions, dyes, and oils in the environmental water systems, many other bio-contents, such as DNA, protein, bacteria, virus, and drugs molecules, exist. Traditional methods, such as chromatography, could be used for the purification of water with bio-contents with high affinity, but the cost is high and not suitable for the high-throughput treatment of wastewater. CNF-based nanomaterials can be fabricated into nanofiltration membranes with various functions for the adsorption and removal of bio-contents with high efficiency, low cost, and good sustainability.

**Figure 9:**
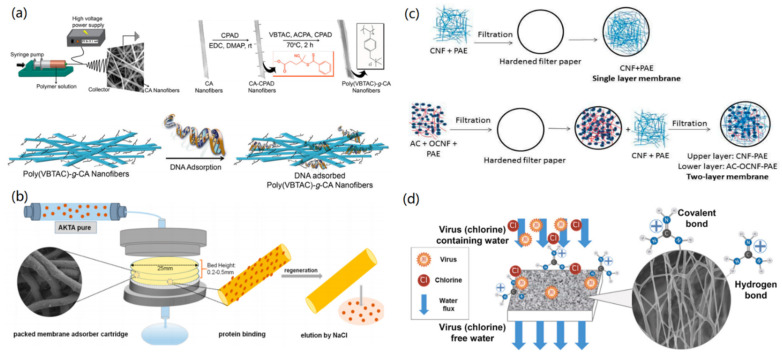
CNF-based functional nanomaterials for the removal of bio-contents: (**a**) Removal of DNA. Reproduced with permission from Ref. [107], Copyright 2014, Elsevier Ltd. (**b**) Removal of protein. Reprinted with permission from Ref. [108], Copyright 2018, American Chemical Society. (**c**) Removal of bacteria. Reprinted with permission from Ref. [109], Copyright 2017, MDPI. (**d**) Removal of virus. Reprinted with permission from Ref. [110], Copyright 2020, Elsevier Ltd.

Previously, Tabuchi and Baba have reported the fabrication of a bacterial CNF-based medium for DNA separation [106]. The fabricated CNF medium had a 3D network structure with double meshes, including 10 nm flexible mesh and 10 µm bacterial cellulose fragments. The designed CNF-based medium exhibited excellent ability for the separation of DNAs with a size from 10 to 15 kbp, which is ascribed to the synergistic effect of the double mesh structure and the stereo effect. In another study, Demirci et al. reported the surface polymer modification of electrospun cellulose acetate (CA) nanofibers for DNA adsorption [107]. As shown in Figure 9a, CA solution in dichloromethane/methanol was electrospun into CA nanofibers, which were further modified with poly(VBTAC) via the reversible addition-fragmentation chain transfer (RAFT) polymerization to form poly(VBTAC)-*g*-CA nanofibers. Due to the electrostatic interaction between cationic polymer and negatively charged DNA molecules, DNA could be adsorbed onto the fabricated nanofibrous membrane with a high adsorption capacity of about 23.51 µg/mg. Their study proves the importance of the surface modification of CNFs for applications in biotechnology.

Protein molecules can also be separated from a water system using the CNF-based nanofiltration membranes. For instance, Rajesh and co-workers demonstrated the fabrication of the CNF-based anion-exchange membranes for the simultaneous removal of proteins and viruses [108]. As shown in Figure 9b, cellulose (CL) was first grafted onto polyethyleeamidoamine (PEAA) to form CL-*g*-PEAA, which was then electrospun into CL-*g*-PEAA nanofiber membranes. Due to the characteristics of anion-exchange and 3D nanolayers, the nanofiber membranes exhibited excellent ion-exchange separation efficiency (239 mg/g) toward bovine serum albumin (BSA). In addition, the formed nanofiltration membranes could reject bead particles with different sizes from 40 to 200 nm, showing their potential for the removal of viruses and microbial agents. Therefore, it was concluded that the separation mechanisms are based on the ion-exchange and size-exclusion effects.

Bacteria and virus contamination in drinking water and natural water systems are serious health hazards, which will result in large economic losses and negative effects on public safety. In this regard, CNF-based nanomaterials have revealed great advantages for the adsorption and removal of various bacteria and viruses [109,110,111,112]. For instance, Hassan et al. reported the fabrication of nanofiltration membranes by using CNFs and active carbon (AC) for the removal of *E. coli* from water [109]. In their study, single-layer and two-layer membranes have been fabricated by the filtration method (Figure 9c). The single-layer membrane was fabricated by the filtration of CNFs and polyamide-amine-epichlorohydrin (PAE) on a hardened filter paper, and for comparison, the two-layer membrane was created by using AC, oxidized CNFs (OCNF), and PAE together. It was found that the two-layer membrane revealed higher water flux than the single-layer membrane due to its higher porosity. Both CNF-based membranes exhibited high rejection efficiencies of 96–99% for the separation of *E. coli* bacteria. In addition, the AC/OCNF/CNF two-layer membrane revealed unique characteristics to inhibit the growth of *E. coli* and *S. aureus* bacteria on the upper surface of the membrane, while the single-layer membrane did not show this property. In another case, Mi and co-workers demonstrated the fabrication of guanidine-modified CNF membranes for the adsorption of virus and chlorine [110]. As presented in Figure 9d, guanidine hydrochloride-modified microcrystalline cellulose (MC) was formed through covalent and hydrogen bonds, which was then mixed with polyvinyl alcohol (PVA) to prepare MG/PVA nanofibers via electrospinning. The water-stable MC/PVA nanofiber membrane was useful for removing four logs of non-enveloped porcine parvovirus and enveloped Sindbis virus. In addition, this nanofiber membrane could achieve 58% removal efficiency toward chlorine.

Furthermore, CNF-based membranes and aerogels could also be utilized for the removal of other bio-contents, such as antibiotics and antibodies [113,114,115].

## 4. Conclusions and Outlooks

In summary, we presented and discussed the fabrication and water purification applications of CNF-based 1D nanocomposites, 2D film/membranes, and 3D hydrogels/aerogels. CNFs can be produced from natural biomass materials through chemical treatment and alternatively can be easily fabricated by the electrospinning method. The created CNFs can serve as excellent templates for the binding and in situ synthesis of various metal and metal oxide NPs. Meanwhile, polymers, QDs, CNTs, and 2D materials can be conjugated onto CNFs to form functional nanocomposites. The hybridization of CNFs with these nanoscale building blocks promoted the mechanical properties of CNF materials and extended their applications in the removal of pollutants.

CNF-based 2D and 3D materials exhibited wider applications and improved performance for the removal of cations, anions, oils, organic dyes, and various bio-contents. 2D CNF films and membranes can be produced by fabricating the CNF suspensions via vacuum filtration, electrospinning, and direct casting. The formed 2D CNF materials displayed a layered, porous, network structure, which are highly beneficial as nanofiltration membranes for water purification. Three-dimensional CNF hydrogels can be constructed by the gelation of CNFs, which was induced by metal ions (such as Fe^3+^, Zn^2+^, Al^3+^), polymers, 3D printing, and direct ink writing. Subsequently, various drying methods, such as freeze-drying, freeze-thawing/ambient drying, and freeze-casting, can be utilized to transfer hydrogels into aerogels.

Here we would like to provide several perspectives on the potential studies in the future. First, economic and sustainable methods should be utilized for the production of CNFs with high efficiency and low cost. Some natural sources and biomass, such as woods, cotton, grass, waste paper, water lily and others, could be potential precursor materials for the production of CNFs. The applications of these natural resources promote the sustainability of both synthesis methods and synthetic materials. Second, CNF-based new materials are still highly required. The hybridizations of CNFs with functional 2D materials, such as MXenes, MOFs, transition metal oxides/dichalcogenides, and black phosphorus, are suggested. Third, the freeze-drying methods for the fabrication of 3D CNF aerogels could be developed to create structure- and function-adjustable materials. Previously, it has been found that the bidirectional, unidirectional, and random freeze casting techniques constructed polymer aerogels with different structures and properties [116]. Fourth, it is necessary to extend the CNF-based functional nanomaterials for air cleaning applications, for instance, the filtering of PM2.5, toxic gases, bacteria, and viruses in the air. Finally, it is necessary to carry out the sustainability evaluation of CNF-based nanomaterials for environmental science applications, which will be helpful for developing CNF-based products for practical high-throughput water treatments, although currently, these applications are still in the laboratory stage.

## Figures and Tables

**Table 1:** Summary of the hybridization, synthesis, and properties of CNF-based 1D, 2D, and 3D nanomaterials.

Dimension	Materials	Synthesis Method	Properties and Applications	Ref.
1D CNF composites	CuO/CNF	Electrospinning, templated synthesis	Antioxidant activity, antibacterial activity	[25]
	Ag/CNF	Thermal treatment and DMF reduction	Antibacterial activity	[26]
	TiO_2_/CNF	Sol-gel synthesis	Photodegradation of methylene blue	[27]
	Ag@Au/CNF	Ionic binding and green synthesis	Catalytic activity, nitrophenol reduction and aza-Michael reactions	[28]
	Cu/CNF and Ag/CNF	Ionic binding and chemical reduction	Antibacterial study	[29]
	Ag/CNF	Green synthesis	Colorimetric sensors	[30]
	Carbon dot/CNF	Microwave synthesis	Colorimetric biosensors	[31]
	BP@/CNF-MOF	In situ solvothermal synthesis	Photocatalytic reduction of U(VI)	[32]
	ZIF-67/CNF	Ionic interaction, templated synthesis	Catalytic activity, energy storage	[33]
	HCNF/CNT	Self-assembly and interface interactions	High strength, high relative humidity, good electrical conductivity	[34]
2D films/membranes	Carboxylic CNFs	Vacuum filtration	High rejection efficiencies (74–80%)	[35]
	CNF/MOF hybrids	Vacuum filtration	CO_2_ and N_2_ separation	[36]
	Ag NWs-Ti_3_C_2_T_x_/CNFs	Vacuum filtration	Electromagnetic interference shielding and thermal management	[37]
	PAN/CNF	Electrospinning	High breaking strength and conductivity	[38]
	PLA/CNF	Electrospinning	Enhanced bioactivity, cell culture	[39]
	Polymer/CNF	Electrospinning and self-assembly	Interlocking multiple membrane layers	[40]
3D hydrogels	CNFs/PAA	Fe^3+^-mediated gelation	High stability, flexibility, conductivity, wearable devices	[47]
	TEMPO-CNFs	Zn^2+^-mediated gelation	Colorimetric indicator of food spoilage	[48]
	P(AMPS-DMA)-CNF	Fe^3+^-mediated gelation	Salt tolerance and thermal stability	[49]
	CNF/alginate	Electrostatic interaction	Tissue engineering and regenerative medicine	[50]
	AL-PEG-CNFs	Self-assembling and -COOH/NH_2_ interaction	Thermo- and pH-responsive	[51]
	PNIPAm/CNF	3D printing	Temperature-sensitive antibacterial properties	[52]
	Aloe vera/CNF	Ink printing	Porosity higher than 80% and a high-water uptake capacity of up to 46 g/g	[53]
3D aerogels	TEMPO-CNFs	Freeze-thawing	Super water absorption and shape memory	[58]
	CNF/xyloglucan	Freeze-casting	Water absorption and shape memory	[59]
	Magnetic CNFs	Freeze-casting	Dry, lightweight, porous, flexible	[60]
	CNF-Si-PEI	Atmospheric drying	Very high specific surface area, and good flexibility	[61]
	CNFs	Freeze-thawing and ambient drying	Highly tensile, transparent, flexible	[62]
	PEI/CNFs	Freeze-drying	Drug delivery	[63]
	BP@CNF-MOF	Freeze-drying, solvothermal synthesis, drying	Photocatalytic reduction of U(IV)	[32]

## Data Availability

Not applicable.
